# The Effect of Information Communication Technology Interventions on Reducing Social Isolation in the Elderly: A Systematic Review

**DOI:** 10.2196/jmir.4596

**Published:** 2016-01-28

**Authors:** Yi-Ru Regina Chen, Peter J Schulz

**Affiliations:** ^1^ Hong Kong Baptist University Kowloon Tong China (Hong Kong); ^2^ University of Lugano Lugano Switzerland

**Keywords:** social isolation, elderly, ICT intervention

## Abstract

**Background:**

The aging of the population is an inexorable change that challenges governments and societies in every developed country. Based on clinical and empirical data, social isolation is found to be prevalent among elderly people, and it has negative consequences on the elderly’s psychological and physical health. Targeting social isolation has become a focus area for policy and practice. Evidence indicates that contemporary information and communication technologies (ICT) have the potential to prevent or reduce the social isolation of elderly people via various mechanisms.

**Objective:**

This systematic review explored the effects of ICT interventions on reducing social isolation of the elderly.

**Methods:**

Relevant electronic databases (PsycINFO, PubMed, MEDLINE, EBSCO, SSCI, Communication Studies: a SAGE Full-Text Collection, Communication & Mass Media Complete, Association for Computing Machinery (ACM) Digital Library, and IEEE Xplore) were systematically searched using a unified strategy to identify quantitative and qualitative studies on the effectiveness of ICT-mediated social isolation interventions for elderly people published in English between 2002 and 2015. Narrative synthesis was performed to interpret the results of the identified studies, and their quality was also appraised.

**Results:**

Twenty-five publications were included in the review. Four of them were evaluated as rigorous research. Most studies measured the effectiveness of ICT by measuring specific dimensions rather than social isolation in general. ICT use was consistently found to affect social support, social connectedness, and social isolation in general positively. The results for loneliness were inconclusive. Even though most were positive, some studies found a nonsignificant or negative impact. More importantly, the positive effect of ICT use on social connectedness and social support seemed to be short-term and did not last for more than six months after the intervention. The results for self-esteem and control over one’s life were consistent but generally nonsignificant. ICT was found to alleviate the elderly’s social isolation through four mechanisms: connecting to the outside world, gaining social support, engaging in activities of interests, and boosting self-confidence.

**Conclusions:**

More well-designed studies that contain a minimum risk of research bias are needed to draw conclusions on the effectiveness of ICT interventions for elderly people in reducing their perceived social isolation as a multidimensional concept. The results of this review suggest that ICT could be an effective tool to tackle social isolation among the elderly. However, it is not suitable for every senior alike. Future research should identify who among elderly people can most benefit from ICT use in reducing social isolation. Research on other types of ICT (eg, mobile phone–based instant messaging apps) should be conducted to promote understanding and practice of ICT-based social-isolation interventions for elderly people.

## Introduction

It is estimated that the proportion of the world population aged 60 years and older will reach 22% by 2050 [1]. Social isolation among the elderly is therefore a growing concern. Depending on the definition and measure, the prevalence of social isolation among people aged 60 years and older is 7% to 24% [2-7] compared to 7% in the general population [6]. In addition, perceived social isolation is more severe among the older old people (aged 75-85 years) than the younger old (aged 57-65 years) [8]. Most importantly, social isolation is a real threat to the mental and physical health of the elderly population [7-11], leading to depression [3,12], self-harming (eg, drug abuse, alcoholism, suicide) [13-15] or self-neglecting behavior [16], a higher level of cognitive and/or physical disability [17], and increased mortality [8,18]. Consequently, preventing or ameliorating social isolation in that age group is becoming a top social topic and a priority in policy-making in many countries [19-20].

Social isolation is a multidimensional concept that lacks a clear and consistent definition in the literature [21-22]. Some scholars see it as directly equivalent to loneliness and use the terms interchangeably [20]; others perceive the two concepts as related yet distinct. For example, social isolation has been defined as the absence of contact with people who provide social support [23]. Others have defined it as a 2-dimensional concept that contains an objective absence of contacts or interactions with the contacts and a subjective feeling of limited or lost companionship or social support (ie, loneliness) resulting from having limited contacts or interactions [8,21]. No matter which definition one adopts, social isolation is considered a result of the elderly population’s reduced social interactions—particularly with family, friends, and community networks—caused by their retirement, physical changes (cognitive and physical disabilities), inevitable loss of spouse or friends (shrinking network size), and/or living alone or in institutions [8]. Information and communication technology (ICT) may overcome the social and spatial barriers of social interaction by enabling easy, affordable communication and activities of multiple forms (ie, textual, audio, and/or visual) between the elderly (often with limited mobilization) and others anytime and anywhere. Many researchers have therefore been investigating its potential for alleviating social isolation in the elderly.

A search of the literature identified 4 systematic reviews [20-21,24-25] that synthesized the effects of social isolation interventions. These reviews examined studies of various designs, including randomized controlled trials (RCTs), experiments, quasi-experimental studies, and before-and-after (cohort) studies, published in the periods 1970 to 2002 [21,24], 1976 to 2009 [20], and 2000 to 2013 [25]. While 3 reviews examined all forms of interventions for social isolation [20-21,24], Morris and colleagues [25] focused only on the interventions using smart technologies to synthesize the effect of interventions on social connectedness of the elderly living at home and found conflicting results.

The objective of our systematic review is to gain a synthesis of the evident effects of ICT interventions on social isolation in the elderly. Our review is timely and valuable for the following reasons: (1) it reviews the effect of ICT interventions on the elderly with various characteristics (eg, demographics, health status, and living arrangements); (2) it covers the most recent research, published between 2002 and 2015; and (3) in addition to quantitative research, it includes studies that used qualitative methods (ie, observations, in-depth interviews, and focus group interviews) to offer insights into the mechanisms underpinning the observed variations in ICT effectiveness.

## Methods

### Searching Strategy, Inclusion Criteria, and Study Selection

Electronic searches for this systematic review were conducted in July 2015 using PsycINFO, PubMed, MEDLINE, EBSCO, SSCI, Communication Studies: a SAGE Full-Text Collection, Communication & Mass Media Complete, Association for Computing Machinery Digital Library, and IEEE Xplore. These databases were used because they include research on subjects such as health, aging, social science, digital technologies, computer-mediated communication, and communication science. A unified search term using Boolean operators was applied for all databases: ((social isolation OR loneliness) AND elderly AND (Internet OR social media OR information and communication technology)). Next, to ensure a broad inclusion of published studies relevant to our review topic, we adopted the following criteria to select studies for the review: (1) publications must be in English; (2) studies must empirically investigate the effects of ICTs on one or more attributes of social isolation among the elderly; and (3) study participants must be aged 55 years or older.

**Figure 1:**
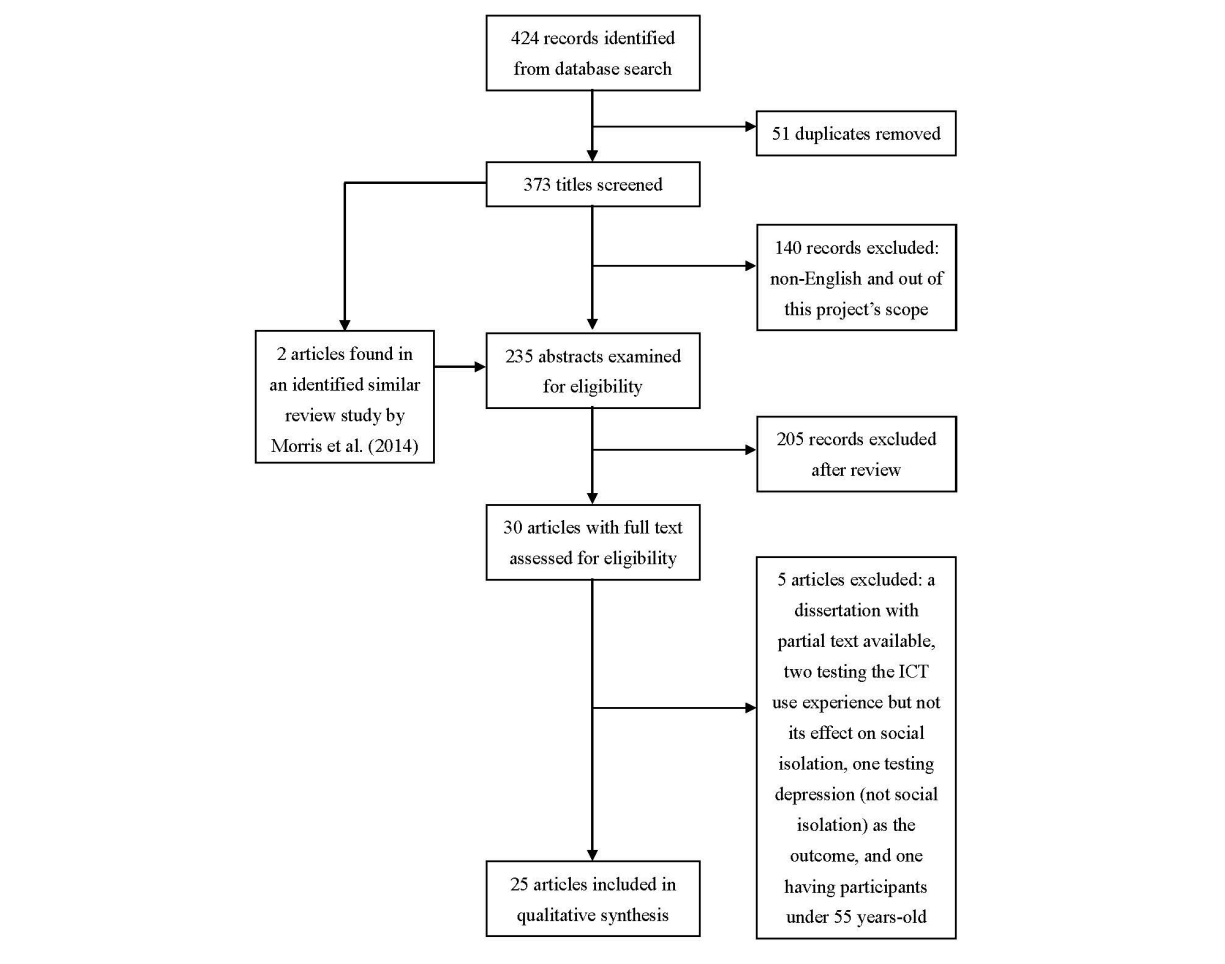
An overview of the inclusion process.

The search yielded 424 publications, of which 51 duplicates were removed. The first author then checked the remaining titles and abstracts to determine their relevance. If the information provided by a title or abstract was insufficient for determination, the full paper was screened by 2 researchers who documented the reasons leading to the exclusion of full texts. An additional 2 studies were found in the systematic review of studies on the elderly population’s social connectedness and smart technologies by Morris et al [25]. A total of 30 articles met the inclusion criteria outlined in Figure 1 and were retained for this systematic review. After carefully reading the full texts of the articles, researchers excluded 5 more studies because of a lack of a complete text (1 article), no examination of social isolation as the outcome of ICT use (3 articles), and the participants being aged younger than 55 years (1 article).

### Data Analysis and Synthesis

Data on study design, sample size and characteristics, types of ICT applications, targets of elderly interaction via ICT applications, comparison groups, and outcomes were extracted from the selected studies and analyzed using a coding scheme. For research quality assessment of quantitative research, the Effective Public Health Practice Project (EPHPP) tool [26] was used because of its suitability for assessing such research with various study designs. The EPHPP tool evaluates 6 components of a quantitative study: selection bias, study design, confounders, blinding, data collection method, and withdrawals and dropouts. Based on performance in each component, an overall rating (ie, strong, moderate, or weak) of each study can be determined. The criteria proposed by Salmon [27] were used to evaluate the qualitative research: theoretical framework, value of study, data collection, participant description, data analysis, and data interpretations. For publications reporting more than one study, each study was independently analyzed. Data coding and quality appraisal were conducted by the first author and a research assistant, reaching an intercoder reliability of .91. Any inconsistencies between the reviewers were discussed between the 2 authors to achieve agreement.

The included studies differed in their research designs, research locations, participant characteristics, types and usage of interventions, and outcome measures. In view of the studies’ heterogeneity, a narrative synthesis (instead of a meta-analysis) was performed [28].

## Results

### Characteristics of Examined Studies

All projects were published between 2002 and 2015, with 11 dated before 2010 [29-39] and 14 dated in or after that year [40-53]. They were conducted in 12 countries (Austria, Canada, Finland, Israel, Netherlands, New Zealand, Norway, Slovenia, Sweden, Taiwan, United Kingdom, and United States) with the highest number coming from the United States (n=9). In the 25 projects, 30 studies were reported (5 projects reported 2 studies: 1 quantitative and 1 qualitative). RCTs comprised 6 studies [36,39,43,47,51-52]; another 6 were cohort studies (2 with a control group [30,35] and 4 without [31,38,41,44]). Of the remaining studies, 4 were cross-sectional studies (surveys) [32,37,40,46] and 14 were qualitative studies: 9 employing in-depth interviews [29-31,34-35,42,45,48-49], 3 conducting focus group interviews [33,38,53], and 2 applying participant observations [39,50]. See Multimedia Appendix 1 for a complete description of the characteristics of the 25 reviewed publications.

Most research used some form of Internet or Web-based apps (eg, search, email, online chat rooms, videoconferencing, social networking apps, and Web-based telehealth systems) on computers. Among those that did not, 1 study employed a telephone befriending intervention, 1 used mobile phones (smartphones), 1 focused on iPad use, 1 applied Nintendo Wii (a video game system), and 1 used a visual pet companion app that allowed the senior users to interact with a pet avatar in real time through an android tablet. The ICT intervention in all but 2 studies was implemented in the regular living environments of the participants, including private housing (n=13), assisted and independent living communities (n=2), congregate housing sites (n=1), retirement villages (n=2), nursing homes (n=4), day care centers (n=1), and no specifics on where they resided (n=2). The intervention was implemented in both settings for 3 projects [35,39,41] that worked with participants from 2 selected residential settings. The visual pet intervention [50] was mobile, and the participants used it in their familiar surroundings not bound by their living environment. The Finland study by Blažun et al [41] had the intervention set up at a community college. The ICT intervention in all but 2 studies aimed to facilitate interaction with other people in general (the participants most frequently contacted their family members, friends, significant others, doctors, and acquaintances made through online chat rooms). The other 2 studies [51-52] designed the intervention for the older person’s interaction with family members only.

### Characteristics of Participants

Sample size of the studies varied from 8 to 5203. The number of participants in the RCT studies ranged from 22 to 205. The sampling strategy of most studies (n=25) was convenience sampling; 3 [36,40,46] used random sampling and 2 [43,48] did not specify the sampling strategy.

Participants’ average age ranged from 66 years (SD not given) to 83 years (SD 1.4) with heterogeneity across demographics, including age, gender, education, income, health status, mental status, living arrangements, and nationalities. Of the chosen studies, 1 [45] had equal numbers of male and female participants and 1 [38] had more males (n=17) than females (n=15). For the remaining studies, female participants often far outnumbered males even though ICT use among the elderly is highly associated with males [54]. There are two possible explanations for this phenomenon: (1) women have longer life expectancy across nations than men and (2) women are more likely to feel lonely and communicate with others and are therefore more likely to participate in such studies. Of the 25 projects, 2 [30,42] recruited living-alone elders only. In terms of participant health characteristics, 4 projects [29,36,41,47] targeted elderly people in generally good health and 5 [30-31,34,38,42,50] looked at those at high risk (lonely, frail, or chronically ill or physically handicapped seniors, those having dementia, and carers of spouses with dementia or after stroke). The other studies did not use health status as a filtering criterion for sampling. For other participant characteristics, 1 study [49] examined elderly former Soviet Union immigrants in Israel with financial difficulties and 1 [33] targeted elderly people interested in computer use. See Multimedia Appendix 2 for details on participant characteristics.

According to the EPHPP quality assessment tool, an attrition rate of 40% or above indicates weak data collection for a study [26]. Based on this standard, 3 studies [30-31,52] were assessed to have a large number of dropouts. Fokkema and Kinpscheer [30] targeted solitary, lonely seniors with chronic illness or physical disability. The participants’ physical and psychological conditions might account for the high attrition rate (43%) even considering the addition of 6 participants from the waiting list to replace the first 8 dropouts. The other 2 studies [31,52] were longitudinal projects lasting 12 months. The study duration contributed to the high rate of participant attrition, especially in a study targeted at the elderly in nursing homes [52]. Of the participants in Mellor’s study [31], 60% were lost in the follow-up. In the study by Tsai et al [52], 44% of the participants in the control group did not complete the study. Of particular concern was a study by Machesney el al [50] in which the number of dropouts was unfortunately not specified but referred to as “several.”

### Dependent Variables and Outcome Measures

The outcome of ICT use was examined in 4 studies [42,43,48,49] by exploring its effect on social isolation in general, while the remaining studies assessed specific aspects of social isolation only. Social isolation as an outcome indicator was only quantitatively measured by Cotton et al [43] using a self-developed scale that contained 3 items, asking how frequently the participant was bothered by (1) not having a close companion, (2) not having enough friends, and (3) not seeing enough of people they feel close to. The other 3 qualitative studies did not clearly define the term. Catton and colleagues [42] seemed to regard social isolation as being forgotten and not belonging. Kahlbaough et al [47] and Karimi and Neustaedter [48] linked the concept to “not being connected with family, friends, and existing contacts.” It should be noted that researchers in the 3 qualitative studies perceived social isolation and loneliness as highly interrelated, if not interchangeable, while Cotton et al [43] analyzed social isolation and loneliness as two separate outcomes of ICT use.

Studies that examined ICT impact on social isolation did so by looking at its effect on 1 or more of the 7 single attributes of social isolation: loneliness, social support, social contact, number of confidants, social connectedness/social connectivity, social networks, and social well-being. Among these, loneliness was the most tested dependent variable (n=18). It was measured by the University of California Los Angeles Loneliness Scale in 20 of the 25 projects. Fokkema and Knipscheer [30] used de Jong-Gierveld and Kamphuis’ loneliness scale [55] whereas Aarts et al [40] used the scale’s short version of 6 items [56]. Heo et al [46] employed the social support scale by Schuster et al [57] to assess loneliness while Sum et al [37] adopted the Social and Emotional Loneliness Scale. Rather than using a standardized scale, Blažun et al [41] used self-reported items of loneliness by the elderly participants in their pre-intervention survey to evaluate outcomes.

Social support was assessed by Tsai and colleagues [51-52] using Hsiung’s Social Support Behaviors Scale, which includes subscales regarding (1) number of social networks, (2) quantity of social support behavior (emotional, informational, instrumental, and appraisal support), and (3) satisfaction with social support. The social support instrument used by Torp et al [38] was adopted from the scale developed by Russel and colleagues [58]. Torp et al also examined social contact as another outcome indicator, applying Andersson’s [59] Family and Friendship Contacts Scale. Social well-being was conceptualized as a multidimensional variable by Slegers et al [36] and was measured using de Jong-Gierveld and Kamphuis’ loneliness scale and the number of social networks. Social connectedness/social connectivity and social networks examined in the reviewed qualitative studies were not clearly defined but were related to the number of connections with others and/or with society at large. Mellor et al [31], however, measured social connectedness using Lee and Robin’s Social Connectedness Scale [60] in their cohort study.

It is worth noting that even though depression is not a dimension of social isolation, it is a related concept that attracts much academic attention. Of the reviewed studies, 6 also examined depression as an outcome variable [35,39,42,50-52]. This research tendency reflects the previous findings that social isolation leads to depression (a negative indicator of psychological well-being) among the elderly. Self-esteem, self-control, and quality of life were the other related outcomes of ICT intervention tested in the studies.

### Effects of ICT Interventions on Alleviating Social Isolation

Of the studies addressing the relationship between ICT usage and social isolation in general, 4 demonstrated a positive result: the use of telephone befriending programs [42], computer and Internet [43,49], and ICT in general [48] lessened social isolation. The reported effect of ICT use on the individual dimensions of social isolation was consistent across studies, except for that on loneliness. ICT interventions significantly fostered social support, social contacts, social connectedness/social connectivity, and social networks among the participants, but no effect was found on number of confidants [39] or social well-being [36].

Of the studies examining loneliness, 15 of 18 revealed a significant reduction of loneliness among the elderly using ICT. Studies using communication programs (using landline phones, smartphones, iPads, emailing, and online chat rooms or forums) and high-technology apps (Wii, the TV gaming system, and Gerijoy, a virtual pet companion) consistently reported a positive effect on alleviating loneliness. The general use of computer and Internet in an RCT design was assessed in 2 nonsignificant-result studies [36,39], with 1 [36] targeting healthy elderly people living at home and the other [39] targeting elderly people living in subsidized housing or nursing facilities. The remaining non-significant study [40] examined the use of social networking sites in particular. Considering that other studies reporting a significant effect of such interventions also used the RCT and survey design targeting the elderly with different levels of health status and in various living situations, it is evident that the effect of the computer and Internet and of social networking sites on improving loneliness among the elderly was inconclusive. Another inconclusive finding concerns the effect of videoconferencing on loneliness reduction among the elderly. Blažun and colleagues [41] found that Slovene participants at nursing homes reported no change of loneliness level after their use of Skype, while loneliness of Taiwanese nursing home participants was significantly lessened after their videoconferencing via Skype or Windows Live Messenger [51-52].

Furthermore, Sum et al [37] found that computer and Internet use functioned differently for various types of loneliness: social loneliness, family loneliness, and romantic loneliness. Using computers and the Internet to communicate with acquaintances alleviated elderly people’s social loneliness, but heavy usage (of long duration) was positively associated with social loneliness. In addition, using the computer and Internet to make new contacts resulted in family loneliness. The impact of computer and Internet use on romantic loneliness was not determined.

Internet use increased social support among the elderly in general [46] and among those who were the main carers of their spouses with dementia or after a stroke in particular [38]. In a similar vein, Nahm’s [32] survey data revealed a positive function of the elderly population’s Internet use in building computer-mediated social networks, which led to social support. Interview data from the study by Dhillon et al [45] suggested that ICT (such as Facebook or networking games) fostered social interaction and social support that further alleviated loneliness among the elderly. Tsai et al [51] found that videoconferencing chats between elderly people at nursing homes and family members significantly increased emotional (ie, caring, empathy, love, and trust) and appraisal (ie, communicating information relevant to self-evaluation) support but not informational (ie, communicating information for problem-solving assistance) or instrumental (ie, tangible goods, services, and aid) support. However, this positive effect on social support was not found at the 6-month or 12-month stages of the intervention [52]. In addition, videoconferencing chats gave lower perceived instrumental support at the 6-month or 12-month stages while the frequency of in-person visits was not changed. The instrumental support finding may, as claimed by the researchers, imply that video chats assisted the elderly in better adapting to the living environment in the nursing home. Thus, their need for tangible goods, services, or aid dropped as their length of residence increased.

The relationship between ICT use and social connectivity/social connectedness or social networks was tested in 6 projects, which reported a generally consistent pattern. ICT in general (Internet, mobile/smartphones, iPads, social networking sites, and audio/video chat apps) served as an effective means for the elderly to remain connected with others [31,35,44,48] and expand their social networks [32,53]. It is important to note that Mellor and colleagues [31] reported that elderly people’s use of computer and Internet at home increased their social connectedness at the 3-month stage of intervention but not at the 6-month or 9-month stage.

In addition to the social-isolation dimensions, a few studies explored the impact of ICT on related constructs, including depression, anxiety, negative affect, cognition, physical functioning (or daily activities), self-control (or perceived control), self-esteem, and quality of life (or life satisfaction). The results pertaining to the effect of ICT use on depression were consistent and generally positive with only 1 study [50] reporting no clear information in the results section. While 2 of the 3 studies concluded that ICT led to positive effects, 1 [31] reported inconclusive results in this outcome. A favorable influence of ICT on life satisfaction was revealed in 4 studies [35,42,47,50], while 2 studies [31,39] found non-significant change in life satisfaction after using ICT. Neither self-esteem [31,39] nor control over life [36,39] was identified as a significant outcome of ICT use. Perception of self-control was, however, significantly increased after accessing ICT [35]. ICT use was found to improve physical health in the elderly [42]. Its effect on increasing the physical activities was inconclusive [36,47].

Lastly, a few studies assessed the effect of ICT use on the quantity and quality of communication of the elderly with others, and 3 studies [39,43,53] found a positive outcome. More elderly participants from Clark’s study [29] stated that the Internet chat environment did not confine their messages for communication than said it did.

### Quality Assessment of Examined Studies

While 4 quantitative studies were rated as moderate [35,43,46,52] and 4 as strong [36,39,47,51], 8 were rated as weak [30-32,37-38,40-41,44]. Among the 4 strong studies, Slegers et al [36] stood out with its rigorously controlled, randomized design. After randomizing twice, it compared the long-term effect of computer and Internet use on loneliness among the elderly in 4 conditions: training-intervention, training-no intervention, no training-no intervention (people in this condition had an interest in computer and Internet use), and control group (people here were not at all interested in ICT use).

The majority of the studies used a convenience sample that resulted in a high risk of selection bias. Consistent with the evaluation results of Morris et al [25], all but 6 of the chosen quantitative studies [36,40,44,49,51-52] failed to specify the proportion of the source population participating or the proportion of those who agreed to participate in the assigned group. The lack of such information makes it hard to determine the samples’ representativeness. Of the 6 studies, 2 [40,44] had a participation proportion of less than 60%. None of the studies report any attempt to blind the participants from the intervention outcomes being examined. Information about whether the assessor (or caregiver) was aware of the intervention was very limited. There were 2 studies [30,43] that did not examine the possible differences between the experiment and control groups prior to the intervention. Furthermore, 3 studies [36,43,50] did not specify the duration of ICT intervention, and in 2 studies [35,43] the format of ICT training (ie, individual or group training) was not reported. Information about the training format is necessary because the literature suggests a relationship between the format and effectiveness of training for the elderly, who are likely to be slow learners of ICTs [31,39].

Of the 16 quantitative studies, 7 controlled for confounding factors in the analyses of effects of ICT interventions. Such factors included number of friends and family [43], physical/emotional/social limitations [43], number of children [35], positive life events [35], personal motivations (ie, learning new skills and gaining attention from others) [36], personality, perceived psychological health [34], length of residency in nursing homes [51-52], educational level [40], sex [40], and age [40,51-52]. Of particular concern was the high percentage of participants who dropped out over the course of the trial in a few studies and the lack of power analysis conducted in all but 1 [36] of the RCT studies.

Among the qualitative studies, 4 [30-31,35,38] were conducted as a secondary analysis to provide further insights into the results of a (randomized) quantitative study. The quality of most qualitative studies was low because the authors failed to address several key areas, as proposed by Salmon [27]. First, most such studies (whether stand-alone or secondary) were descriptive or exploratory without examining specified propositions derived from the literature, while 3 studies [33,49,53] discussed the findings based on theories. Second, the interviewee recruitment processes were not clearly specified. Most studies reported the sampling frame and characteristics of the interviewees but some failed to provide information about the recruitment method (eg, randomly, purposively, or conveniently recruited) and others did not explain how a certain location, nursing home, or community was selected and why. Additionally, even though most studies reported the number of interviewees, none of them mentioned whether the number was a result of theoretical saturation. Without such information, readers are unable to determine the appropriateness and richness of the data. Furthermore, most studies [30-31,34-35,39,48,50] did not clearly report how the data were collected and analyzed. They often failed to report the interview protocol or the coding procedure even though some did state how they identified the emerging themes. This information is crucial because the researchers’ approach to the data directly determines what the findings are. Lastly, a serious concern was that many authors reported the data superficially without interpretation or implications.

## Discussion

This systematic review is, to our knowledge, the first to address the potential of ICT for preventing or reducing social isolation, a state that implies the risk of deteriorating physical and psychological well-being for the elderly. The results of this systematic review provide emerging quantitative and qualitative evidence to support the function of ICT in alleviating social isolation (in general or in particular dimensions) among elderly people. This review advances the mechanism of how ICT assists the elderly in combating social isolation and provides insights for policies and practices.

### Social Isolation as an Untested Concept

Most studies of the review evaluated the effect of ICT use on single social-isolation dimensions, including loneliness, social support, and social connectedness. This pattern is consistent with that revealed in the review of studies by Dickens and colleagues [20] on social isolation interventions for the elderly, where only 2 of the 32 studies used social isolation as an outcome variable while the remaining studies mostly assessed loneliness, social network size, and social support. These findings suggest that social isolation of the elderly as a multidimensional concept is largely understudied [20,22]. Even though evidence shows that the use of ICT affects specific aspects of social isolation, its effect on the overall perception of social isolation remains largely unknown. Therefore, more research is needed to unlock the relationship between ICT interventions and social isolation reduction.

The limited examination of the general concept of social isolation as a multidimensional construct might be a result of the lack of an appropriate scale. Sansoni et al [61] found the following 4 instruments of social isolation to be the leading ones in the literature: the Lubben Social Network Scale [62-64], the de Jong-Gierveld Loneliness Scale [55-56], the Medical Outcomes Study Social Support Survey [65], and the Multidimensional Scale of Perceived Social Support [66]. These instruments are clearly designed for measuring particular aspects rather than the overall concept of social isolation. Future research is required to develop a reliable scale of social isolation as a multidimensional variable by first discovering the concept’s underpinnings from the perspective of the elderly. For instance, the comprehensive review of social isolation literature conducted by Nicholson [22] identified 5 key attributes of social isolation: (1) belonging, (2) social contacts, (3) quality of relationships, (4) fulfilling relationships, and/or (5) engagement. When investigating the complex relations between social isolation and health, Cornwell and Waite [8] operationalized social isolation as a variable of multiple components (ie, social contact frequency, social network size, social activity, loneliness, and social support) integrated into 2 forms: social disconnectedness and isolation. Further evaluation should be performed to validate the applicability of the instruments for measuring single aspects of social isolation versus that of the instruments tapping social isolation as a multidimensional construct. Even though some dimensions of social isolation were addressed by the studies included in this review, there are still some such as quality of relationships and engagement that remain untested in relation to the effect of ICT intervention. Researchers should explore these dimensions in future studies to advance our understanding of social isolation.

### The Mechanism of ICT in Alleviating Social Isolation

ICT use consistently affected social isolation in general, social support, and social connectedness positively, but the positive ICT effect on social connectedness and social support rarely lasted for more than 6 months after the intervention. The results for loneliness were inconclusive. The results for self-esteem and control over life were consistently nonsignificant.

After triangulating the quantitative and qualitative data of the included studies in this review, it is suggested that the elderly’s employment of ICT reduces their social isolation through the following mechanisms: connecting to the outside world, gaining social support, engaging in activities of interest, and boosting self-confidence. ICT helps the elderly stay connected with their family members (especially grandchildren), friends, former colleagues, acquaintances, and new contacts of shared interests or needs across temporal and geographical boundaries via digital interactions. Connections lead to social inclusion and foster social support. ICT also allows elderly people to renew their hobbies or competence and participate in enjoyable activities without the time constraint. Most importantly, ICT use boosts self-confidence among the elderly by making them “connected to information,” “feel young,” “become one of the modern generation,” “overcome challenges,” “equip themselves with new skills,” “stay socially active,” and “help others online.” It is worth noting that providing advice to the younger generation (acquainted or unknown) has a significant positive impact on the elderly population’s self-confidence. The self-confidence gained leads to self-efficacy that goes beyond the use of ICT and participating in social activities. ICT use also empowers the elderly by engaging them in critical thinking and decision-making and providing access to information and resources. Self-confidence and empowerment further trigger their positive feelings toward themselves and their control over life and/or life satisfaction. Thus, a further examination of self-efficacy, mastery, and empowerment as outcomes should be promising for theory building in the field of social isolation.

### ICT Use Among the Elderly

The findings of this review suggest that the elderly can benefit from ICT interventions and will use them (sometimes frequently) after proper training. At the same time, the high attrition rate of participants in the trials and the inconclusive results of ICT impact on loneliness reduction imply that ICT is not suitable for every senior. Spatial (eg, home-bound or institutionalized) and social (eg, immigrants or spousal carers) barriers to socialization, interest in ICT, motivations for ICT use, cognitive capability, sufficient eyesight, and basic physical ability to use the equipment (eg, figure or hand movement, skills of using the touch pad) are possible predictors of the suitability of ICT for the elderly. Furthermore, tailor-made training for the elderly (in terms of its setting, procedure, materials, timing, and instructor’s style and attitude) is necessary for a maximum positive effect of the ICT on alleviating social isolation.

There are different mechanisms by which ICTs influence different kinds of loneliness and social support among the elderly [37,51-52]. The results reveal the interplay between the ICT-mediated activity and the effect of such behavior on particular types of loneliness and social support. Considering that there were only 2 studies addressing the types of loneliness and 2 examining types of social support, future research on these topics should advance the understanding of ICT’s role in alleviating social isolation. Results of such research can provide insights into which individuals among the elderly can most benefit from ICT to reduce their loneliness or increase their social support in particular cases.

### Future Development

The majority of the reviewed studies tested the ICT intervention as a one-time trial among a small number of participants. Thus, the generalizability of the results is limited. Further examination is needed to test their applicability.

Most ICT interventions examined in this systematic review involved the use of the computer and Internet. With the rapid development of ICT, other types of interventions should be explored. As stated by some interviewed participants of the reviewed studies, the use of ICT allowed them to adjust to their younger family members;#8217 communication style and preferences. As a result, it enhanced the quantity and quality of their intergenerational communication. Similarly, Clark [27] observed that use of a particular platform, if one has a sufficient number of friends, lessens social isolation. ICT that is currently prevalent—instant messaging (eg, WhatsApp, Line, Snapchat), YouTube videos, and social networking sites (eg, Facebook, Instagram)—should be further investigated for the potential in reducing social isolation among the elderly. For example, Harley and Fitzpatrick [67] found that YouTube allowed a senior user to engage in communication beyond the family context with younger YouTubers who shared his interests using self-made videos (ie, videoblogging). Such behavior further fulfilled the senior’s social and emotional needs and increased his self-confidence. Also of interest for further research are mobile phone apps, because elderly people demonstrate a fast-growing rate of mobile phone-based ICT adoption across their age groups in wealthy countries [68].

Additionally, the results of this review suggest that ICT use does not guarantee quality of communication. For example, when the ICT-mediated communication is not reciprocal, the ICT use could increase social isolation among the elderly [48]. Consequently, examining how to use ICT for generating quality communication between the elderly and others (eg, using videoconferencing for the elderly to virtually join family activities) can be a promising subject for future research on social isolation and/or intergenerational communication.

### Strengths and Weaknesses of the Review

This systematic review tackled an emerging trend of social isolation research: ICT interventions for reducing social isolation in the elderly. The comprehensive search strategy and the inclusion of studies of all designs increased the likelihood of including all relevant studies in the field. Presenting the results of both randomized and nonrandomized research might be a limitation of this study. However, this review decision broadened our exploration of the available social-isolation interventions and their effectiveness and helped to better achieve the objective of this study.

The heterogeneity of studies included in this review limits the comparability and generalizability of our results. Although restricting the scope to studies published in English might introduce bias, the reviewed studies were conducted in America, Europe, and the Asia-Pacific region.

### Conclusion

This systematic review has suggested a need for more well-designed studies on the effect of ICT interventions on the social isolation of elderly people. ICT in general is a promising tool for tackling social isolation of the elderly, but it is not for every senior. Research identifying who among the elderly can most benefit from ICT use and how the training and implementation of such intervention should be tailored to maximize its effect offers great value for clinical practice. In addition, with the rapid development of ICT, the effectiveness of other types of interventions (eg, mobile phone-based instant messaging apps and YouTube videos) in reducing social isolation should be empirically examined. Results of such research can facilitate innovative and effective practice of ICT-based social isolation interventions for elderly people.

## Appendix

Multimedia Appendix 1 Characteristics of the selected studies.

Multimedia Appendix 2 Participant characteristics of the selected studies.

## Abbreviations

EPHPP: Effective Public Health Practice Project
ICT: information and communication technology
RCT: randomized controlled trial
