# Supplementary material for: The Effect of Information Communication Technology Interventions on Reducing Social Isolation in the Elderly: A Systematic Review
Source: J Med Internet Res. 2016 Jan 28;18(1):e18. doi: 10.2196/jmir.4596 (PMC4751336; doi:10.2196/jmir.4596)
Supplement: Multimedia Appendix 1 [file jmir_v18i1e18_app1.pdf]

Study (year)	Research design	Intervention (ICT description)	Intervention usage	Dependent variables	Outcomes measures	ICT Impacts on outcomes and effect sizes	EPHPP rating
Aarts et al. (2014)	Cross-sectional study	Social networking sites (SNS)	Unrestricted	Loneliness (emotional, social, & in general)	6-item de Jong-Gierveld Loneliness Scale (2006)	SNS usage was unrelated to emotional loneliness, social loneliness, & loneliness in general	Weak
Blažun et al. (2012)	Cohort study w/o a control group	Computer, 3 weeks of group training (12 hours in Finland, 9 hours in Slovenia)	Unrestricted	Loneliness	Self-reporting items by the elderly participants	The elderly who do not use Skype to communicate with friends and send up to 5 emails per week had a decreased level of loneliness	Weak
Cattan et al. (2011)	In-depth interview	Telephone befriending service, no training provided	Unrestricted	Social isolation, loneliness, depression	NA	Reduction of social isolation, loneliness, depression, and anxiety; improved state of mind, contentment with life, confidence level, and physical health (less pain)	NA
Clark (2002)	In-depth interview	Internet-at-home	Unrestricted (reported average use: 26hrs/week on computer & 27hrs/week on Internet)	Loneliness	NA	Reduced loneliness (70% of the participants reported it)	NA
Cotten et al. (2013)	RCT	Internet-at-home, 8 weeks of training (training format not specified)	Unrestricted (reported average use: 1.3 times/ month)	Social isolation, loneliness, social network, social interaction	Self-developed social isolation scale with 3 items, UCLALS (3 items), 7 questions for the quality of online social interaction	Decreased loneliness ($\beta = .172$, $P = .001$); less isolated ($\beta = .491$, $P < .001$); more connected ($\beta = .392$, $P = .001$); earlier to make new contacts ($\beta = .297$, $P = .01$); better communication quality ($\beta = .289$, $P = .010$)	Moderate
Delello & McWhorter (2015)	Cohort study w/o a control group	iPad, 3 group training of 1.5-hour lessons over 6 weeks	Unrestricted	Social connectedness	NA	iPad training increased its use by the elderly that further fostered their connection with family, online community, and existing friends	Weak

Note. n.d. = not documented, NA = not applicable.

Study (year)	Research design	Intervention (ICT description)	Intervention usage	Dependent variables	Outcomes measures	ICT Impacts on outcomes and effect sizes	EPHPP rating
Dhillon et al. (2011)	In-depth interview	Web-based telehealth system, no training provided	Unrestricted (reported use: daily use of the system)	Loneliness, socialization	NA	Social support via Facebook-like features and networked games that trigger social interactions could reduce loneliness, motivate each other, and share experiences	NA
Fokkema & Kinpscheer (2007)	Cohort study w/a control group	Internet-at-home, 5 lessons of 2-hour training at home	Unrestricted for 3 years	Loneliness	de Jong-Gierveld & Kamphuis (1985) Loneliness Scale	Loneliness reduction after 2 years (*P* = .050) and 3 years (*P* = .02) of intervention Significant loneliness decrease among the well-educated elderly in 2 years (*P* = .04) and 3 years (*P* = .008) of intervention	Weak
	In-depth interview			Effect of ICT on loneliness & its cause	NA	3 mechanisms of ICT to cope with loneliness	NA
Heo et al. (2015)	Cross-sectional study	Internet	Unrestricted	Social support, loneliness	Schuster et al. (1990) social support scale Hughes et al. (2004) loneliness scale	Internet use led to social support ($\beta = .096$, $t = 6.051$, $P < .001$) that reduced loneliness ($\beta = -.139$, $t = -8.034$, $P < .001$)	Moderate
Kahlbaugh et al. (2011)	RCT	Wii, no training provided	1 hour/week for 10 weeks	Loneliness, affect, physical activity, life satisfaction	UCLALS	Lower loneliness ($P < .005$) in the Wii group compared with the control group	Strong
Karimi & Neustaedter (2012)	In-depth interview	Internet, mobile phones; social media (e.g. Facebook), video chat systems (e.g., Skype)	Unrestricted	Social connectivity, loneliness, social isolation	NA	Adaption to ICTs helped stay connected and reduce loneliness and social isolation	NA

Note. n.d. = not documented, NA = not applicable.

Study (year)	Research design	Intervention (ICT description)	Intervention usage	Dependent variables	Outcomes measures	ICT Impacts on outcomes and effect sizes	EPHPP rating
Khvorostianov et al. (2011)	In-depth interview	Internet	Unrestricted	Social isolation, loneliness	NA	Elderly immigrants used Internet to maintain existing contacts and expand social networks that reduced their social isolation and loneliness. Internet use for entertainment and nurturing new hobbies also alleviated loneliness and hard feelings in life.	NA
Machesney et al. (2014)	Observation	Gerijoy (a virtual pet companion application), no training provided	Unrestricted, use length not documented	Loneliness; depression cognition, self-esteem, quality of life	NA	Reduced loneliness (no effect of other outcomes but life quality was reported)	NA
Mellor et al. (2008)	Cohort study w/o a control group	Internet-at-home , individual training on a daily basis in the project's first 2 weeks	Unrestricted for 12 months	Social connectedness; Self-esteem, quality of life, positive affect	Social connectedness scale (Lee & Robins, 1995)	Social connectedness & self-esteem increased after 3 months but declined at the 6, 9, 12 months of Internet use; positive affect was up & down	Weak
	In-depth interview			Internet use impact on social isolation; frequency, experience, & barriers of Internet use	NA	Internet use had a positive impact on social connectedness	NA
Nahm et al. (2003)	Cross-sectional study	Internet-at-home	Unrestricted (reported average use: 4.23 ± 0.9 years)	Social support, computer-mediated social networks, psychological well-being	n.d.	Usage of Internet & email and PC knowledge enlarged the participant's computer-mediated social networks (CMSN) that increased social support; age & physical barrier of PC use led to a smaller CMSN; no effect size reported	Weak
Richardson et al. (2005)	Focus-group interview	Internet-at-home	Unrestricted	Social connectedness	NA	Elderly used computer to connect to others, to information, and to the modern world.	NA

Note. n.d. = not documented, NA = not applicable.

Study (year)	Research design	Intervention (ICT description)	Intervention usage	Dependent variables	Outcomes measures	ICT Impacts on outcomes and effect sizes	EPHPP rating
Savolainen et al. (2008)	In-depth interview	Videoconferencing system	Unrestricted (reported average use: 5.7 initiated calls & 40 minutes of call time per month)	Loneliness	NA	7 out of the 8 participants reported a positive impact on loneliness	NA
Shapira et al. (2007)	Cohort study w/a control group	Internet at nursing homes, 1 or 2 1-hour training sessions for 15 weeks (training format not specified)	Unrestricted for 15 weeks	Loneliness; depression, life satisfaction, self-control, physical functioning (PF)	Revised UCLALS, depressive objective checklist	Loneliness was significantly lower in the Internet group compared with the control group [F(1, 33) = 34.71, *P* < .001, η^2 = .051], effect on other outcomes was sig. but not on PF	Moderate
	In-depth interview			Dynamics of the tested variables	NA	Learning of ICT, social benefits of online communication, involvement & action, positive feelings by ICT use	NA
Slegers et al. (2008)	RCT	Internet-at-home, three 4-hour group training sessions for 2 weeks	Unrestricted, use length not documented	Social wellbeing, emotional wellbeing, physical wellbeing, belief in external control, control over life, instrumental activities of living	Loneliness questionnaire (de Jong-Gierveld & Kamphuis, 1986), nature & frequency of social networks	No significant effects found	Strong
Sum et al. (2008)	Cross-sectional study	Internet-at-home	Unrestricted (reported use: 96.8% of the participants used Internet for more than a year, and 44% for more than 7 years)	Loneliness	Social and Emotional Loneliness Scale (SELSA)	Using Internet for communication led to lower social loneliness (β = -.124, *P* = .002). Internet usage for new contacts led to family loneliness (β = .145, *P* = .005); long Internet usage time was also positively linked to social loneliness (β = .151, *P* = .002)	Weak

Note. n.d. = not documented, NA = not applicable.

Study (year)	Research design	Intervention (ICT description)	Intervention usage	Dependent variables	Outcomes measures	ICT Impacts on outcomes and effect sizes	EPHPP rating
Torp et al. (2008)	Cohort study w/o a control group	PC with internet & online discussion forum, three 3-hour group training sessions over 3 weeks	Unrestricted for 12 months	Social contact, social support	Family and Friendship Contacts Scale (Andersson, 1984),Russel et al. (1980) social support scale	ICT use increased social contacts [Change M = 1.5, CI for mean (0.06-2.88), *P* = .04] and social support [Change M = 3.4, CI for mean (1.14-5.61), *P* = .010]	Weak
	Focus-group interview (at 7 months)				NA	ICT use most facilitated contacts with grandchildren. It also led to better mood, new and supportive friends with same experience, and away from cared-for-person. Negative outcomes: much time spent on ICT	NA
Tsai et al. (2010)	RCT	Videoconferencing interaction, a trained research assistant spent at least 5 minute per week with each participant at the time of use	Once a week for 3 months	Social support, loneliness, depression	Social support behavior scale (Hsiung, 1999), UCLALS, Geriatric Depression Scale	Emotional & appraisal social support was higher in the intervention group at 1 week ($\beta = .42$, $P < .001$; $\beta = .39$, $P = .001$) & 3 months ($\beta = .61$, $p < .001$; $\beta = .68$, $P < .001$); loneliness was lower in the intervention group at 1 week ($\beta = -1.21$, $P = .002$) & 3 months ($\beta = -2.84$, $P = .003$)	Strong

Note. n.d. = not documented, NA = not applicable.

Study (year)	Research design	Intervention (ICT description)	Intervention usage	Dependent variables	Outcomes measures	ICT Impacts on outcomes and effect sizes	EPHPP rating
White et al. (2002)	RCT	Internet-at-home, 9 hours of group training	Unrestricted for 5 months	Loneliness, depression, perceived control, attitude toward computer, life satisfaction, confidant size	UCLALS, the CES Depression Scale, Perceived Control Scale, Attitudes Toward Computers Scale, life satisfaction, number of confidants	No significant effects found (only a trend of less loneliness, less depression, and more number of confidents)	Strong
	Observation			Goals, difficulties, challenges, and benefits of computer use		Positive impact of computer use on reducing loneliness, depression & boredom in life; such use was a rewarding challenge	NA
Winstead et al. (2012)	Focus-group interview	Internet at AICs, 29 hours of group training sessions over 8 weeks	Unrestricted for 8 weeks	Social networks, familial communica-tion	NA	ICT use led to the online-community participation, renewed prior relationships, and enlarged familial communication of AIC residents. But the effect size varied by AIC characteristics.	NA

Note. n.d. = not documented, NA = not applicable.
