# Supplementary material for: The Effect of Information Communication Technology Interventions on Reducing Social Isolation in the Elderly: A Systematic Review
Source: J Med Internet Res. 2016 Jan 28;18(1):e18. doi: 10.2196/jmir.4596 (PMC4751336; doi:10.2196/jmir.4596)
Supplement: Multimedia Appendix 2 [file jmir_v18i1e18_app2.pdf]

Study (year)	Setting	Sampling	Sample character	Size		Dropouts	Age	
				Total	Gender (M, F)		Mean (SD)	Range
Aarts et al. (2014) Netherlands	Home	Random	No specifics	626	(310,316)	NA	n.d.	60+
Blažun et al. (2012) Finland (F) & Slovenia (S)	F: community college S: nursing homes	Convenience	F: no specifics S: healthy	F: 17 S: 28	F: (47.7%, 52.3%) S: (33.9%, 66.1%)	F: 10 S: 3	F: 66.34 (6.01) S: 77.36 (8.24)	n.d.
Cattan et al. (2011) UK	Where they reside	Convenience	Vulnerable , isolated, and/or lonely (most living alone)	40	n.d.	NA	n.d.	mid-50s to early 90s
Clark (2002) US	Where they reside	Convenience	Healthy (4 excellent in health, 4 good, 2 fair)	10	(3, 7)	1	67.8 (n.d.)	65-73
Cotten et al. (2013) US	Assisted & independe nt living communiti es (AICs)	n.d.	n.d.	205 (trail: 79, control: 126)	(36, 169)	0	82.8 (7.7)	n.d.
Delello & McWhorter (2015) US	Retirement village	Convenience	n.d.	19	(3, 16)	n.d.	n.d.	61-99
Dhillon et al. (2011) New Zealand	Home	Convenience	3 living alone and 5 living with family	8	(4, 4)	NA	72 (n.d.)	60-87
Fokkema & Kinpscheer (2007) Netherlands	Home	Convenience	Chronicall y ill, physically handicappe d, lonely, & living alone	Cohort study: 35 (trial: 21, control: 14); Interview: 4	Trial: (1, 11), control: (7, 7); interview : n.d.	9 (cohort study)	Trial: 66 (n.d.), control: 68 (n.d.); interview : n.d.	n.d.
Heo et al. (2015) US	Home	Random (2008 US Health and Retirement Study data)	No specifics	5203	(41%, 59%)	NA	77.87 (8.06)	65-105
Kahlbaugh et al. (2011) US	Home	Convenience	Healthy	36	(4, 32)	1	82 (9.8)	n.d.

Note. n.d. = not documented, NA = not applicable.

Study (year)	Setting	Sampling	Sample character	Size		Dropouts	Age	
				Total	Gender (M, F)		Mean (SD)	Range
Karimi & Neustaedter (2012) Canada	Home	n.d.	n.d.	12	n.d.	NA	n.d.	55-83
Khvorostianov et al. (2011) Israel	Home	Convenience	Healthy	32	(17, 15)	NA	76 (n.d.)	69-89
Machesney et al. (2014) US	Participants' familiar surroundings	Convenience	Dementia patients	13	(4, 9)	several	n.d.	65-93
Mellor et al. (2008) Australia	3 retirement villages	Convenience		Cohort: 20; interview: n.d.	(1, 19); interview: n.d.	3 (at 6 months), 7 (at 9 months), 12 (at 1 year)	75.92 (7.61); interview: n.d.	55-88
Nahm et al. (2003) US	Home	Convenience	n.d.	809	(511, 751)	NA	67.8 (n.d.)	55+
Richardson et al. (2005) New Zealand	Home	Convenience	Interested in computer learning	98	(43, 55)	NA	n.d.	55-88
Savolainen et al. (2008) Sweden	Home	Convenience	frail	8	(1 , 7)	0	n.d.	mid-50s to early 90s
Shapira et al. (2007) Israel	Nursing homes and day care centers	Convenience	n.d.	48 (trial: 22, control: 26); interview: 7	Trial: (9, 13), control: (9, 17); interview: n.d.	9 (trial: 6, control: 3)	Trial: 80.25 (6.50), control: 82.6 (5.9); interview: n.d.	Both groups: 70-93; interview: n.d.
Slegers et al. (2008) Netherlands	Home	Random	Healthy	204 (training-trail: 60, training-no trial: 49, no training-no trail: 55, control: 40)	n.d.	25 in total at 4 months, 32 at 12 months	n.d.	64-75
Sum et al. (2008) Australia	Home	Convenience	n.d.	222	(38%, 62%)	NA	n.d.	55+
Torp et al. (2008) Norway	Home	Convenience	Carers of an ill spouse	19	(11, 8)	1	73 (n.d.); interview: n.d.	57-85

Note. n.d. = not documented, NA = not applicable.

Study (year)	Setting	Sampling	Sample character	Size		Dropouts	Age	
				Total	Gender (M, F)		Mean (SD)	Range
Tsai et al. (2010) Taiwan	Nursing homes	Cluster, convenience	n.d.	57 (trial: 24, control: 33)	Trial: (18, 22), control: (20,30)	8 (trial: 3, control: 5)	Trial: 73.82 (11.19), control: 79.26 (7.07)	60+
Tsai & Tsai (2011) Taiwan	Nursing homes	Cluster, convenience	n.d.	90 (trial: 40, control: 50)	Trial: (10,14), control: (14, 19)	35 (trial: 13, control: 22)	Trial: 74.42 (10.18), control: 78.48 (6.75)	60+
White et al. (2002) US	4 congregate housing sites and 2 nursing facilities	Convenience	n.d.	100 (trial: 51, control: 49); observation: n.d.	Trial: (29%, 71%), control: (18%, 82%); observation: n.d.	16 (trial: 12, control: 4)	Trial: 73.82 (11.19), control: 79.26 (7.07); observation: n.d.	n.d.
Winstead et al. (2012) US	3 AICs	Convenience	n.d.	43	(9, 34)	8	83.0 (1.4)	n.d.

Note. n.d. = not documented, NA = not applicable.
